# A Rest Quality Metric Using a Cluster-Based Analysis of Accelerometer Data and Correlation With Digital Medicine Ingestion Data: Algorithm Development

**DOI:** 10.2196/17993

**Published:** 2021-03-02

**Authors:** Zahra Heidary, Jeffrey M Cochran, Timothy Peters-Strickland, Jonathan Knights

**Affiliations:** 1 Otsuka Pharmaceutical Development & Commercialization, Inc Princeton, NJ United States

**Keywords:** serious mental illness, rest quality, actimetry, behavioral health, digital medicine, accelerometer, medication adherence

## Abstract

**Background:**

Adherence to medication regimens and patient rest are two important factors in the well-being of patients with serious mental illness. Both of these behaviors are traditionally difficult to record objectively in unsupervised populations.

**Objective:**

A digital medicine system that provides objective time-stamped medication ingestion records was used by patients with serious mental illness. Accelerometer data from the digital medicine system was used to assess rest quality and thus allow for investigation into correlations between rest and medication ingestion.

**Methods:**

Longest daily rest periods were identified and then evaluated using a k-means clustering algorithm and distance metric to quantify the relative quality of patient rest during these periods. This accelerometer-derived quality-of-rest metric, along with other accepted metrics of rest quality, such as duration and start time of the longest rest periods, was compared to the objective medication ingestion records. Overall medication adherence classification based on rest features was not performed due to a lack of patients with poor adherence in the sample population.

**Results:**

Explorations of the relationship between these rest metrics and ingestion did seem to indicate that patients with poor adherence experienced relatively low quality of rest; however, patients with better adherence did not necessarily exhibit consistent rest quality. This sample did not contain sufficient patients with poor adherence to draw more robust correlations between rest quality and ingestion behavior. The correlation of temporal outliers in these rest metrics with daily outliers in ingestion time was also explored.

**Conclusions:**

This result demonstrates the ability of digital medicine systems to quantify patient rest quality, providing a framework for further work to expand the participant population, compare these rest metrics to gold-standard sleep measurements, and correlate these digital medicine biomarkers with objective medication ingestion data.

## Introduction

Lack of adherence to medication regimens is a significant public health issue that contributes to increased health care utilization [1,2]. Adherence is of particular concern in patients with serious mental illness (SMI), including schizophrenia, bipolar disorder, and major depressive disorder, with estimates of nonadherence as high as 60% [1,3]. Within this population, effective pharmacotherapy is critical for mitigating the risk of serious adverse events, such as psychosis, symptom recurrence, poor social functions, hospitalizations, and suicide attempts [4,5]. Conventional methods of inferring medication adherence to pharmacotherapy have limited utility, as they are typically either subjective or acquire only surrogate markers of medication ingestion [6]. Thus, there is a clear, unmet clinical need for adherence monitoring that digital medicine is ideally suited to address. In this context, digital medicine refers to a system that combines an active pharmaceutical and an ingestible sensor that communicates to a mobile app or web-based application to record that patients have taken their medication at a specific time [7], providing an objective measure of ingestion.

Another potentially useful biomarker for patients with SMI is disruption in sleep [8,9] and activity patterns [10,11], both of which have been significantly linked to mental health. The digital medicine system (DMS) used here noninvasively records activity-related parameters such as step count, physical patient orientation, and heart rate. Due to battery optimization for ingestion monitoring, the DMS does not record these metrics at a sufficiently high temporal resolution to perform common sleep identification techniques, such as heart rate variability analysis [12,13]. However, accelerometer-based actigraphy has been extensively validated against gold-standard sleep measurements like polysomnography [10,14-17]; thus, the available data from the DMS can noninvasively provide valuable insight into patterns of rest and activity in patients with SMI at the currently available temporal resolution and battery consumption while simultaneously recording previously unavailable objective medication ingestion data with a single device in a natural care setting.

Thus, the goals of this study were twofold. First, 3-axis accelerometer data from the DMS and a novel analysis algorithm were used to identify and analyze patients’ longest period of rest during each day they were on the system. The relative quality of these rest periods was quantified using a modified k-means clustering algorithm of accelerometer-derived features; previous actimetry research has correlated these types of activity features with wakefulness in sleep studies [14-17]. These activity features should thus also be correlated with measurements of rest quality as defined by the National Sleep Foundation, such as sleep efficiency and wake after sleep onset [18]. The stability of the rest period duration and consistency of the rest period starting time were also assessed across all days for each patient, as measurements of rest duration [18-20] and starting time [9,10,18] have also been correlated with quality of rest. Finally, preliminary correlations between rest and medication ingestion records were explored.

## Methods

### DMS

**Figure 1:**
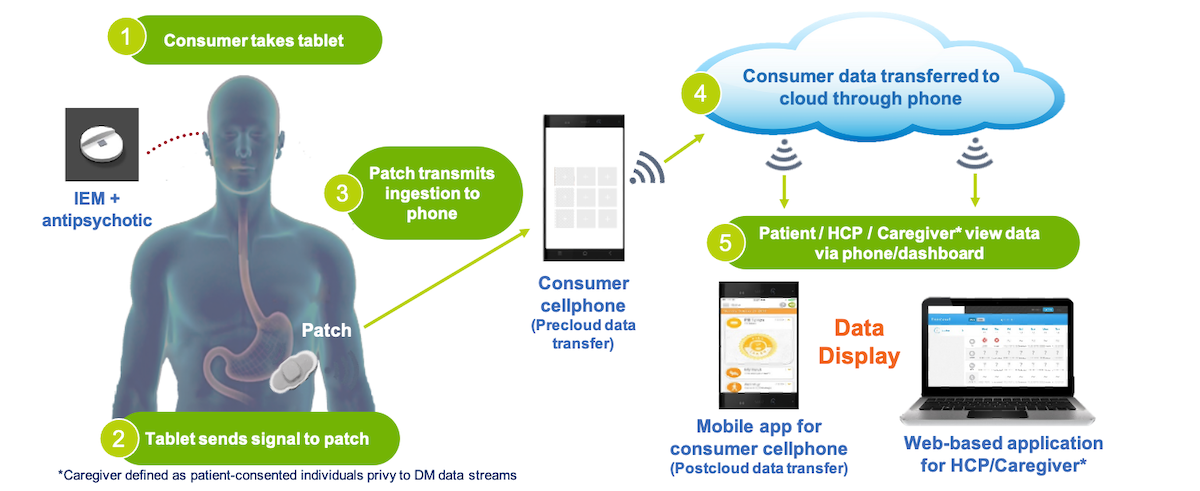
Schematic of the DMS. The DMS consists of an ingestible sensor embedded within an aripiprazole tablet. Upon ingestion, this sensor communicates with a patient-worn patch, which records the ingestion event and provides measures of activity and heart rate using an accelerometer and ECG, respectively, as well as temperature and impedance across the patch. The patch then communicates all data to a smartphone app, which stores the data on a secure cloud server that can be accessed by patients or designated caregivers via a mobile or web-based dashboard. DM: digital medicine; DMS: digital medicine system; ECG: electrocardiogram; HCP: health care provider; IEM: ingestible event marker.

The DMS used here has been described previously [21]. Briefly, the DMS is a combination of an ingestible sensor embedded in an active pharmaceutical and a sensor patch that is attached to the torso, which records ingestion events and measures activity via a 3-axis accelerometer and an estimated sample heart rate with a single-lead electrocardiogram (ECG). The patch, which the patient must apply, also contains a temperature sensor and an impedance sensor and is designed to be worn for 7 days. The collected data is uploaded and stored in the cloud via a mobile phone app that also displays a patient dashboard (see Figure 1).

This system is designed for monitoring medication ingestion in patients with SMI. Each measured ingestion event is recorded with a timestamp. The accelerometer data are measured across a 14-second sample every minute, and a built-in algorithm converts the raw data to a step count, mean acceleration along all 3 axes (a_x_, a_y_, a_z_), and mean body orientation angle (θ), which are read out at 1-minute intervals. When the patch initially observes activity, the accelerometer is calibrated such that the x-axis is approximately oriented along the longitudinal axis of the body regardless of the orientation of the patch. The ECG provides the mean heart rate every 5 minutes.

### Study Population

The population used in this analysis comprised 102 participants enrolled across 2 clinical studies (NCT02219009, NCT02722967) [22,23]. Table 1 contains the demographic and clinical characteristics for these patients. All participants were previously diagnosed with schizophrenia, bipolar disorder, or major depressive disorder and were regularly receiving once-daily doses of the atypical antipsychotic aripiprazole. For the duration of the study, patients received the digital version of aripiprazole. Both studies were reviewed and approved by the appropriate institutional review boards, and patients were all deemed capable of using the DMS and provided signed informed consent. To ensure the reliability of individual rest metrics, the data set presented here excludes 14 additional patients enrolled in the studies that did not have at least 7 days of recorded data from the DMS patch.

**Table 1:** Demographic and clinical characteristics of the sample population.

Characteristic		Values (N=102)
**Gender, n (%)**		
	Female	40 (39)
	Male	62 (61)
Age (years), mean (SD)		45.9 (11.3)
**Race, n (%)**		
	Black or African American	56 (55)
	White	39 (38)
	Asian	5 (5)
	Other	2 (2)
**Ethnicity, n (%)**		
	Hispanic or Latino	5 (5)
	Not Hispanic or Latino	97 (95)
**Diagnosis, n (%)**		
	Schizophrenia	71 (70)
	Bipolar 1 disorder	21 (21)
	Major depressive disorder	10 (10)
**Observed ingestion (%)**		
	Median (IQR)	71.3 (28.9)
	Range	14.8-96.6
**Observed ingestion duration (days)**		
	Median (IQR)	54 (14)
	Range	7-64

### Definition of Longest Rest Periods

The DMS patch provides step count and posture angle measurements from the accelerometer every minute and the mean heart rate from the ECG every 5 minutes. These measurements were partitioned into 15-minute nonoverlapping time intervals, which were assessed for data quality. A 15-minute interval was considered analyzable if all 3 of the following conditions were met: (1) there was no point in the interval at which the patch was pairing with the mobile app, (2) there were at least 10 collected accelerometer measurements within the interval, (3) there were at least 2 ECG records within the window.

Any intervals that failed 1 or more of these criteria were labelled as missing, ensuring that intervals were only used if they contained sufficient reliable patch data.

For analyzable intervals, each 1-minute accelerometer record was then examined, and if the posture angle of that record was less than 30° away from horizontal, it was classified as rest.

Based on these accelerometer measurement labels, the analyzable 15-minute intervals were then classified as either rest (if greater than 70% of the records in that interval were classified as rest) or active (if the rest criterion was not met).

**Figure 2:**
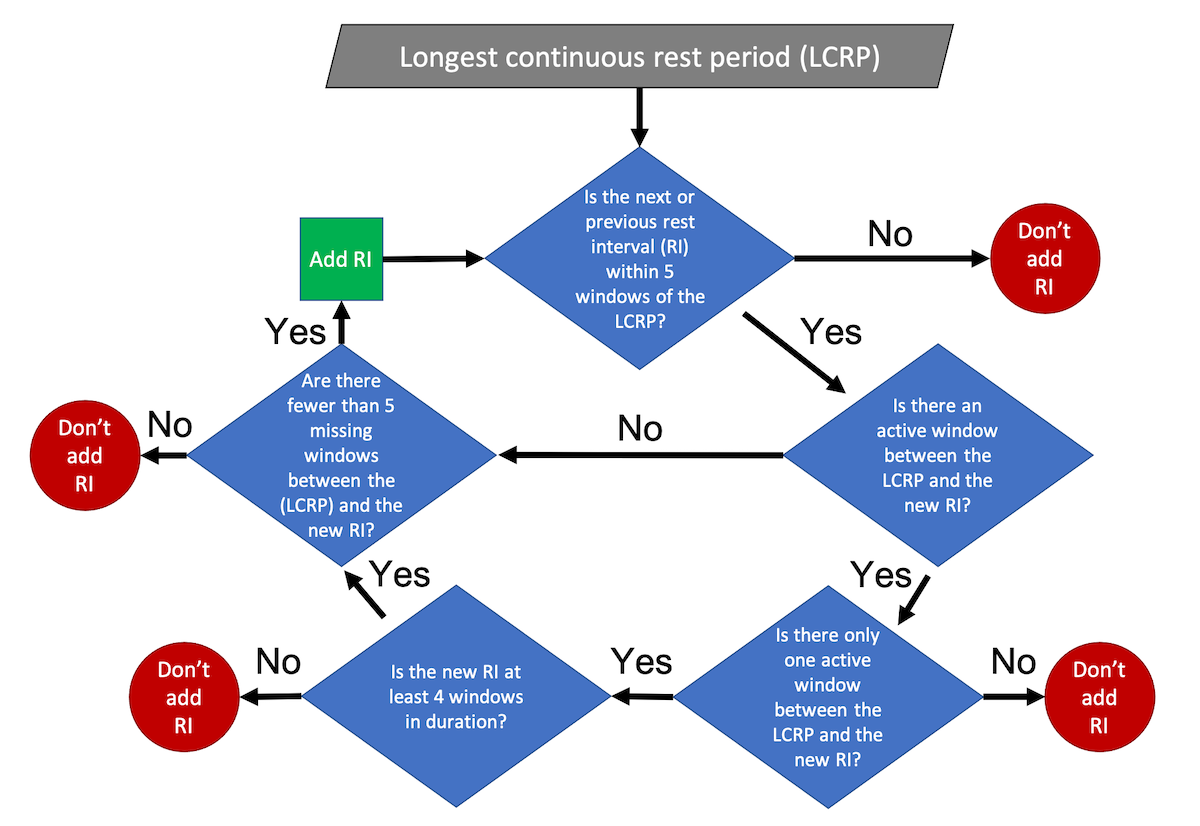
Flowchart for finding the longest rest period. The first step in defining the longest rest period for a given day is to locate the LCRP (ie, the longest duration of consecutive 15-minute rest windows without any active or missing windows). The algorithm described in the flowchart is then used to determine if nearby windows are added to the LCRP.

These 15-minute windows were then used to define each patient’s longest period of rest for a given day. The first step was to identify the longest period of consecutive rest intervals that did not contain any active or missing intervals; however, this simple longest continuous rest period (LCRP) was likely an insufficient representation of the patient’s true longest rest period (LRP). For one, intervals labeled as missing could artificially shorten these periods. Additionally, a single active interval is not necessarily indicative of the end of that rest period; for example, sleep is often interrupted by a short period of activity. Thus, an algorithm was used, as illustrated in Figure 2, to extend these longest periods of rest to more accurately capture the duration of each rest period.

**Figure 3:**
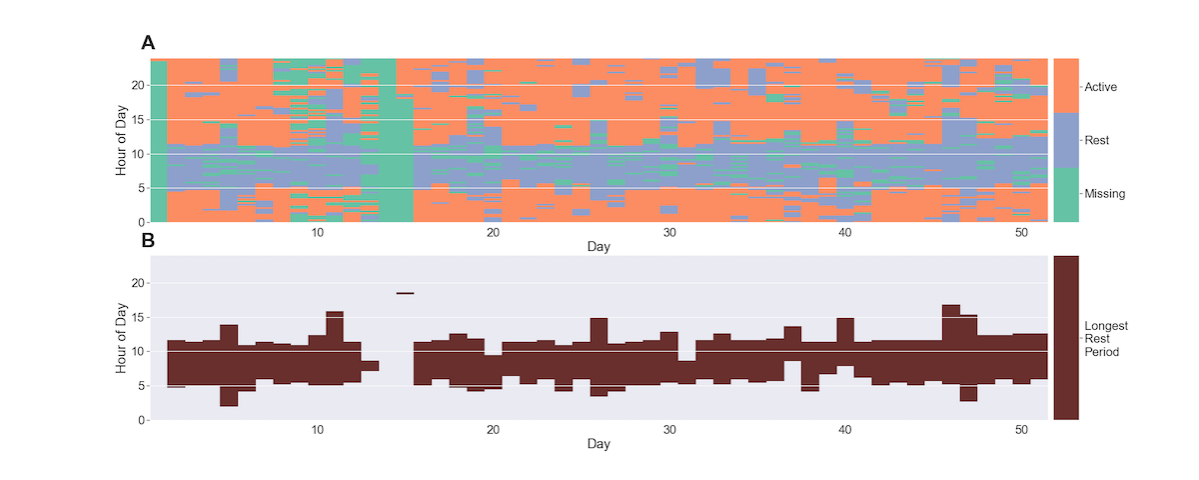
Rest state interval designations and longest rest periods for a single subject. (A) All 15-minute windows were classified as either rest, active, or missing, according to the procedure discussed in the Methods section. (B) The longest rest period for each day was identified using the procedure shown in Figure 2.

Briefly, sequences of continuous rest windows that were within 5 intervals of either the beginning or end of the initial longest LCRP were added to the LRP if the intervening intervals were all missing (ie, none of them were labeled as active). If exactly 1 active interval was present between the longest interval and another rest interval in any of the 5-interval windows, only sequences that contained 4 or more consecutive rest intervals were added to the LRP. If more than 2 intervals between the original period and the additional rest period were labeled as active, the new rest period was not added to the LRP. Figure 3 provides the 15-minute interval rest designations and identified LRPs for a single patient across all days.

### Rest Features and Metrics

With the LRP identified for each patient on each day, features were then developed to quantify the relative quality of rest within these intervals. For this purpose, 3-minute rolling windows of the accelerometer data, consisting of 3D acceleration (a_x_, a_y_, a_z_), posture angle (θ), and step count, were used, resulting in 13 individual data points for each 15-minute window. In all, 35 features (see Multimedia Appendix 1 Table S1) were tested, and 4 features were chosen via a feature agglomeration technique which is similar to agglomeration clustering but uses recursive merging of features instead of samples [24]. The feature agglomeration clustering algorithm was run independently for each patient in the data set, and 4 features that were consistently grouped into separate clusters across all patients were chosen.

**Figure 4:**
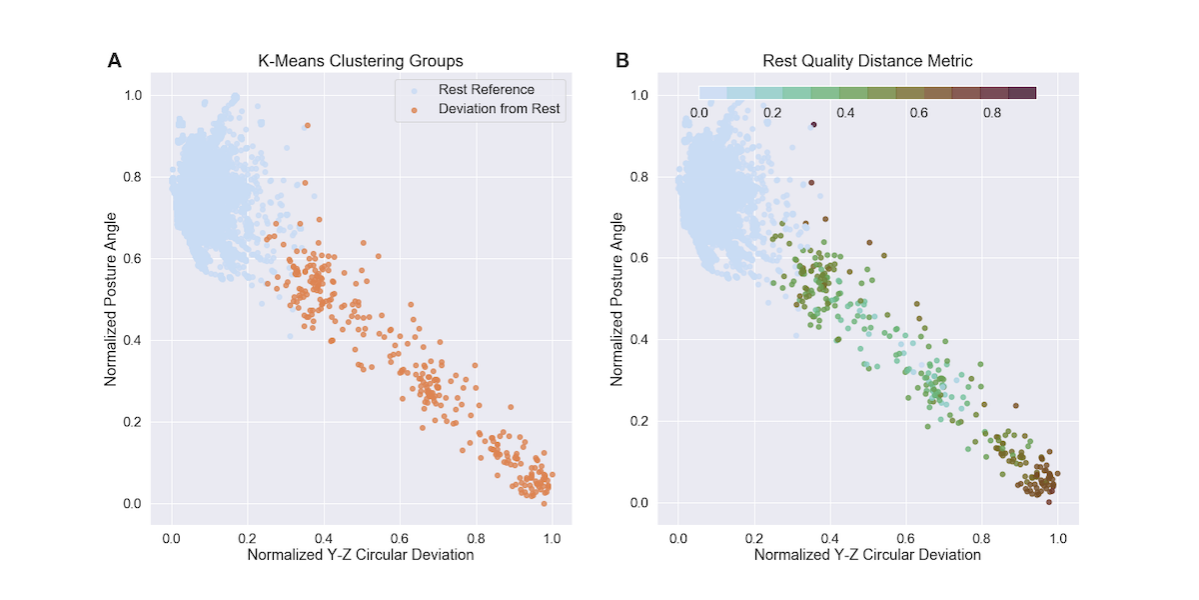
K-means clustering and relative rest quality in a single patient for 2 of the 4 features. (A) K-means clustering diagram plotted across 2 dimensions representing 2 of the 4 features in the clustering algorithm. All data points for each patient were classified using 2 clusters defined as the rest reference cluster (in blue) and the deviation from rest cluster (in orange). (B) Relative rest quality distance metric calculation. A quality of rest metric, represented here by variations in color, was also determined for each data point by calculating the Euclidean distance of the point from the center of the rest reference cluster. All points within the rest reference cluster were assigned a distance of 0. Note that the rest quality color does not have a perfect correlation with apparent distance on this 2D graph because the Euclidean distance is calculated across all 4 feature dimensions.

These 4 features were then normalized to their respective ranges and used in a k-means clustering algorithm with 2 groups (k=2); 2 groups were purposely chosen to identify both a rest-reference (RR) cluster, in which the mean posture angle was closer to a horizontal position, and a deviation-from-rest (DFR) cluster. A Euclidean distance across the 4D feature space quantified the deviation from the rest cluster for each data point; because the 4 individual features used in this model are associated with rest quality, this distance can be interpreted as a metric for the quality of rest. For all points in the DFR cluster, the distance was calculated from that point to the center of the RR cluster in the 4D feature space. All points that were designated as part of the RR cluster were given a distance measure of 0; thus, low values indicated higher rest quality, while higher values indicated poorer rest quality. Figure 4 shows a sample patient cluster diagram with the Euclidean distance calculation.

**Figure 5:**
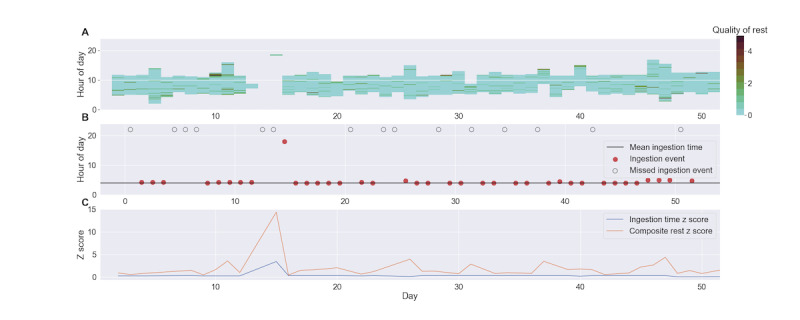
Daily relative rest quality and ingestion for a single patient. (A) The longest rest period for each day. Each bar represents the time windows that constitute the longest rest period for a given day. The color of each 15-minute interval within each bar represents the rest quality metric for that window. (B) Ingestion times for each day. Recorded ingestion events are marked with a red circle, missed ingestions are marked with an empty black circle, and the mean ingestion time across all days is represented by the horizontal line. (C) Ingestion and composite rest z scores. The composite rest z score (in orange), which is the sum of the rest quality, starting time, and duration z score, and the ingestion time z score (in blue) are plotted across all days for the same patient. We searched for data points where outliers in the composite rest z score occurred near the same day as a corresponding outlier in the ingestion time z score (eg, on day 15). Note that days without recorded patch data were excluded from the analysis.

Within each 15-minute window, the distance measurements of all points were then summed to create a single rest quality metric. Figure 5A displays these LRPs and the calculated rest quality in each window for a single patient. The relative rest quality for each LRP was defined as the mean of the relative rest quality in all 15-minute windows within that LRP. Finally, for a given patient, the mean and SD of the relative rest quality of the LRP across all days were used to transform the rest quality metric for each LRP into a *z* score.

The *z* score metrics of the stability of the LRP duration and of the consistency of the LRP starting time were similarly calculated across all days for each participant. A composite rest *z* score for each LRP could then be calculated by summing the absolute values of the quality, stability, and consistency *z* score measurements.

### Ingestion Metrics

**Figure 6:**
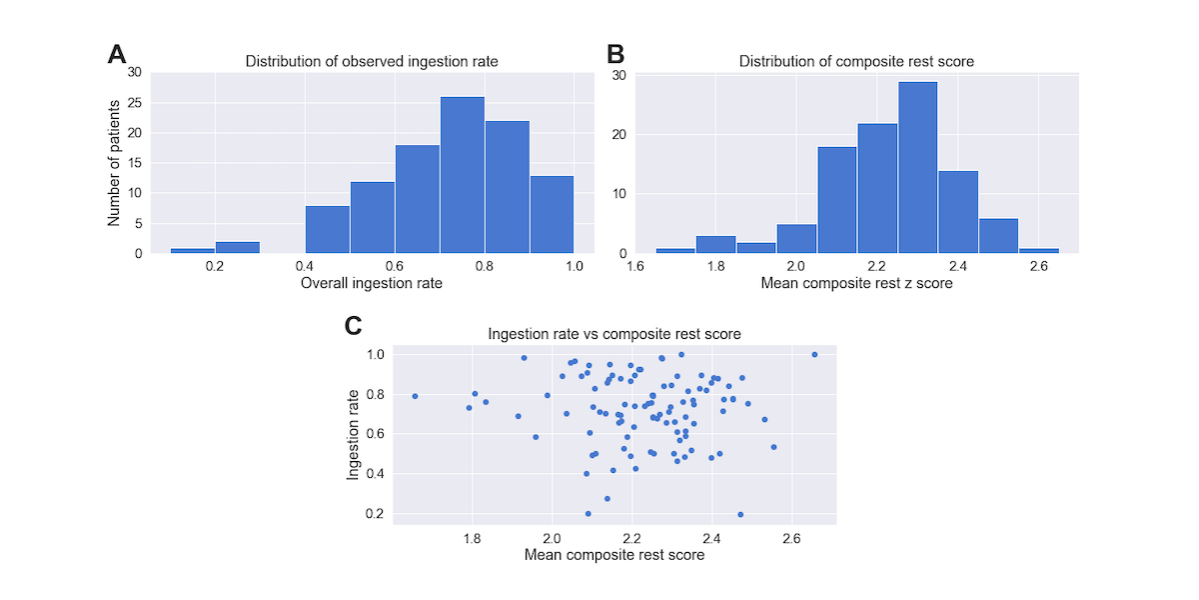
Ingestion rate and composite rest score. (A) Distribution of ingestion rate by patient. Ingestion rate was defined as the fraction of days that contained an ingestion event. Notice that there are relatively few patients with a poor ingestion rate, which made developing robust classification algorithms difficult. (B) Distribution of mean composite rest score across all days for each patient. (C) Ingestion rate versus mean composite rest score. There is no clear correlation between the ingestion rate and the composite rest score. Note, however, that there are no patients with very poor adherence (ie, an ingestion rate less than 0.5) that have a low composite rest score, which would be indicative of better rest.

Patient medication ingestion was quantified with 2 metrics. The first was the patient’s overall observed ingestion rate, calculated as the number of days during which an ingested dose was recorded divided by the expected number of ingested doses across the entire regimen, which was defined as the time from the first day with recorded patch data to the last day with an ingestion record (see Table 1 and Figure 6A). The second metric was a *z* score of daily ingestion time using the mean and SD of ingestion time across all days for a given patient. These ingestion time markers were compared to the previously described rest metrics by searching for single-day outliers in the rest *z* score within 1 day of similar outliers in the ingestion time *z* score (see Figure 5B).

## Results

**Figure 7:**
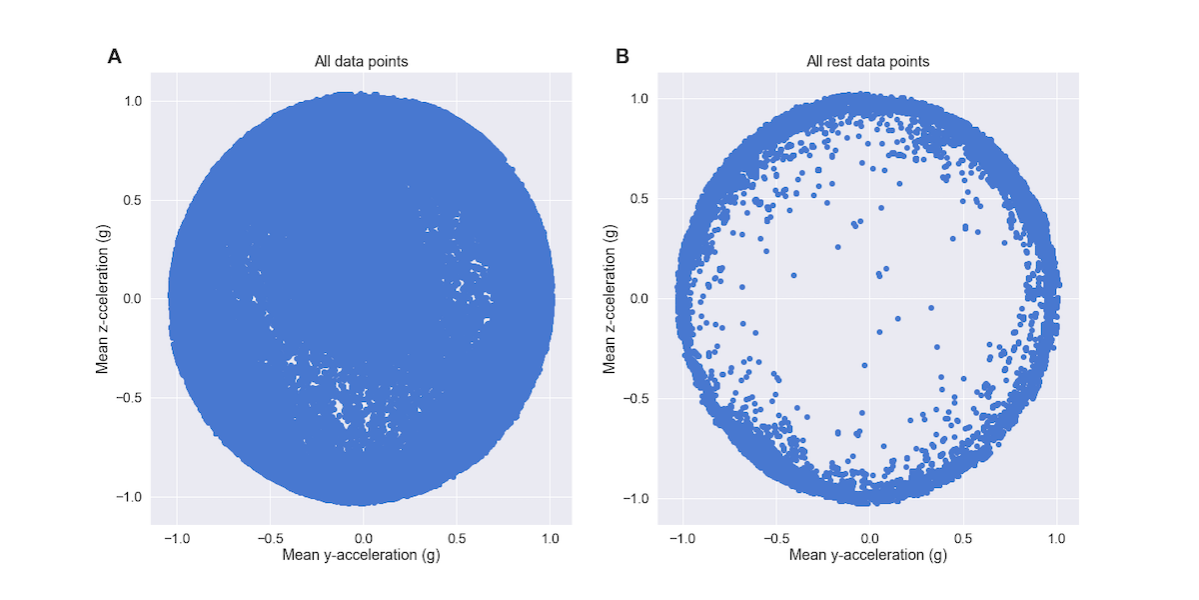
The y–z acceleration circular deviation. (A) Mean z-axis acceleration versus mean y-axis acceleration for all data points in units of g. (B) Mean z-axis acceleration versus mean y-axis acceleration for all rest data points in units of g. Note that, for the remaining data points, the y-axis and z-axis accelerations tend to cluster around a circle with radius 1. Thus, the circular deviation in the y–z plane is an effective feature for differentiating rest from nonrest.

A total of 35 features, calculated across 3-minute rolling windows, were explored to characterize rest in this population of 102 patients with SMI. There were no significant observed differences across the 3 indications in this population (ie, schizophrenia, bipolar disorder, or major depressive disorder) for any of the forthcoming metrics. The 4 features chosen by the aforementioned agglomeration technique were the mean of the circular deviation in the y–z acceleration plane, the mean of the posture angle, the mean of the 3D acceleration norm, and the SD of the x acceleration (see Table 2), which were all calculated across 3-minute rolling windows. The circular deviation in the y–z acceleration, which represents the degree to which the combination of the y and z components of the acceleration differ from the full 1-g resting acceleration, was particularly successful at differentiating rest windows from those designated as active (see Figure 7).

**Table 2:** Rest features.

Name	Definition
y–z acceleration circular deviation (mean)	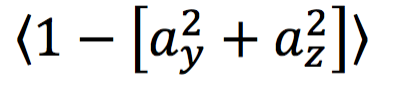
Posture angle (mean)	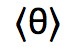
Acceleration norm (mean)	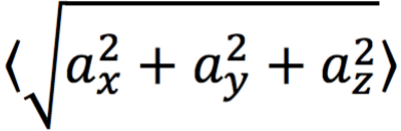
x acceleration (SD)	σ(α_χ_)

These 4 features were used in the k-means clustering algorithm to create the RR and DFR groups as well as the relative rest quality distance metric (see Figure 4). The distance metrics for each 3-minute window were summed over each 15-minute interval, and the mean across the LRP was calculated for each patient on each day, providing a metric of rest quality throughout each LRP (see Figure 5A). Note that the value of this metric is inversely related to the quality of rest. The start time and duration of each LRP was also calculated. The *z* scores for this rest quality, along with the start time stability and duration of the LRPs, were calculated with respect to the mean and SD of all LRPs within a given patient’s data set. A composite rest score was then defined by summing the absolute value of the 3 *z* score metrics for each LRP. This composite score was thus a measure of the combined magnitude of deviation from a patient’s typical pattern for these 3 rest metrics. The distribution of the mean value of this composite rest score for all patients can be seen in Figure 6B.

These rest metrics were then compared to the overall ingestion rate for each patient, which was defined as the fraction of days a patient was using the DMS that contained an ingestion event. This study population had a notable lack of patients with poor adherence; for example, only 15% of patients had an observed ingestion percentage of 50% or less (see Figure 6A). Patients with lower adherence did appear to have a relatively low quality of rest as quantified by the composite rest metric; there were no patients with an ingestion rate equal to or less than 50% that had a low composite rest *z* score (see Figure 6C), which signifies better rest. However, the asymmetry in high versus low adherence and the small sample size precluded statistically significant conclusions about the overall correlation between low adherence and rest quality.

We were, however, able to explore correlations between daily outliers in the composite rest score with outliers in ingestion time that occurred within 1 day of the rest outlier. One such example can be seen in Figure 5C. Although this sample does not contain sufficient events of this type to develop quantitative conclusions, this could be a potentially useful marker for identifying rest-related adherence risk factors in the time domain.

## Discussion

### Principal Results

This DMS combines objective, time-stamped records of medication ingestion with noninvasive physiological markers of activity and heart rate in patients with SMI. It thus provides a unique opportunity to measure the quality of rest and explore correlations between rest and objective medication ingestion data. Here, algorithms were developed to both identify rest periods and quantify the relative quality of the rest within these periods using only the data available from the DMS without increasing battery consumption.

The LRP identification algorithm used accelerometer data, as provided by the DMS, to first find 15-minute windows that could be classified as rest and then to combine these windows to find a daily period of longest rest. Thirty-five potential accelerometer-measured features were explored, and the four best were selected via a feature agglomeration methodology (see Table 2). These features included the acceleration norm and SD of the x-axis acceleration, with larger values associated with greater activity. Another feature was the posture angle, with 0 corresponding to a patient lying down and deviations representing a greater degree of uprightness. The most novel feature was the circular deviation of the y–z acceleration, which is also correlated with rest. If a patient were lying still and perfectly horizontally, the x-axis (ie, head-to-toe) acceleration would be small, and thus the total y–z magnitude of acceleration would be near 1. This would be an example of high-quality rest and result in a y–z circular deviation near 0 (see Figure 4). Larger y–z circular deviations can thus be interpreted as an indicator of decreased rest quality.

These 4 features served as the input for an unsupervised k-means clustering algorithm (k=2) that included a 4D Euclidean distance metric for each data point to the center of the RR cluster, which was then applied to all windows in each LRP. This distance metric thus could quantify the degree to which a patient deviates from the RR cluster. Because each of the 4 features that constitute this distance can individually be correlated with rest quality, this distance metric can thus be interpreted as a deviation from quality rest. All patients used in this clustering algorithm were examined to ensure that their data points were not well represented by a single rest cluster. It should be noted that other populations could contain such a patient, which could minimize the contrast between the RR and DFR clusters. This algorithm demonstrates the ability of the DMS to quantify patient-specific rest quality despite the relatively low temporal resolution of the available accelerometer data due to the need to prioritize power consumption of the ingestion detection module. Thus, rest quality can be quantified for patients already engaged with the DMS without the use of an additional activity tracker that would require further patient engagement.

Exploratory correlations between the calculated rest metrics and ingestion data were hindered by the sample’s asymmetry in medication adherence, in which few patients exhibited poor ingestion rates. Thus, no statistically significant conclusions about rest and overall adherence were drawn. Patients with poor adherence did tend to have larger composite rest *z* scores, which is indicative of inconsistent rest quality; however, patients that were largely adherent to the regimen did not necessarily have more consistent rest as measured by the composite rest *z* score. A *z* score–based comparison of daily outliers in rest and ingestion outliers within 1 day could also serve as a useful metric for exploring the time-domain prediction of variability in medication ingestion time in a study with a larger sample.

### Limitations

Thus, this analysis is an important first step in leveraging the available data from this DMS system to quantify personalized rest metrics for eventual correlation with objective ingestion data, providing insight into the behavioral context of medication adherence for patients with SMI. However, the study does have several limitations that can be addressed with future research. The most notable is the lack of patients with very poor adherence, which prevented the use of rest data to truly classify patients by their adherence. This shortcoming can be easily addressed by accruing more participants in future studies. It would also be interesting to apply these rest metrics to patients in a controlled sleep study, which would enable an assessment of the degree to which these markers for rest are an accurate surrogate of sleep. Finally, an inherent limitation of the DMS is that it provides accelerometer data at only 1-minute intervals. This study nonetheless demonstrates that this relatively sparse data can still be used to effectively monitor patient rest.

There are many other future analysis directions that could be pursued with this data. Two of the most direct extensions of this study would be to more fully incorporate the system’s measurement of heart rate and to begin exploring markers that quantify a patient’s activity level throughout the day.

### Conclusions

Data from the accelerometer in a DMS that provides objective time-stamped medication ingestion records were collected and used to develop novel algorithms for identifying and assessing the quality of daily rest periods for individual patients. A lack of patients with poor adherence in the sample population prevented the use of a quantitative classifier; however, pilot explorations of the relationship between these rest metrics and ingestion provided several insights. Patients with poor adherence experienced lower quality of rest, while patients with high adherence did not exhibit a consistent rest pattern. Additionally, the correlation of outliers in composite rest score with outliers in ingestion time is an interesting potential application of this algorithm. This study is an important first demonstration of the ability of the DMS to track patient rest, which provides a framework for future correlation of DMS-based biomarkers with medication adherence in patients with SMI.

## Appendix

Multimedia Appendix 1 List of all accelerometer features used.

## Abbreviations

DFR: deviation from rest
DMS: digital medicine system
ECG: electrocardiogram
LCRP: longest continuous rest period
LRP: longest rest period
RR: rest reference
SMI: serious mental illness
