# Supplementary material for: A Rest Quality Metric Using a Cluster-Based Analysis of Accelerometer Data and Correlation With Digital Medicine Ingestion Data: Algorithm Development
Source: JMIR Form Res. 2021 Mar 2;5(3):e17993. doi: 10.2196/17993 (PMC7967235; doi:10.2196/17993)
Supplement: Multimedia Appendix 1 [file formative_v5i3e17993_app1.docx]

| **Name** | **Definition** |
| --- | --- |
|  |  |
| **Single-Point Features** |  |
| X Acceleration | $a_{x}$ |
| Y Acceleration | $a_{y}$ |
| Z Acceleration | $a_{z}$ |
| Acceleration Norm | $\sqrt{{a_{x}}^{2}+{a_{y}}^{2}+{a_{z}}^{2}}$ |
| X-Y Acceleration Ratio | $\left\langle\frac{a_{x}}{a_{y}} \right\rangle$ |
| X-Z Acceleration Ratio | $\left\langle\frac{a_{x}}{a_{z}} \right\rangle$ |
| Y-Z Acceleration Ratio | $\left\langle\frac{a_{y}}{a_{z}} \right\rangle$ |
| X-Y Acceleration Circular Deviation | $1-\left[ {a_{x}}^{2}+{a_{y}}^{2} \right]$ |
| X-Z Acceleration Circular Deviation | $1-\left[ {a_{x}}^{2}+{a_{z}}^{2} \right]$ |
| Y-Z Acceleration Circular Deviation | $1-\left[ {a_{y}}^{2}+{a_{z}}^{2} \right]$ |
| Posture Angle | $\theta$ |
| Step Count | $s$ |
|  |  |
| **Three-Minute Rolling Window Features** |  |
| X Acceleration – Mean | $\left\langle a_{x} \right\rangle$ |
| Y Acceleration – Mean | $\left\langle a_{y} \right\rangle$ |
| Z Acceleration – Mean | $\left\langle a_{z} \right\rangle$ |
| X Acceleration – Standard Deviation | $\sigma\left( a_{x} \right)$ |
| Y Acceleration – Standard Deviation | $\sigma\left( a_{y} \right)$ |
| Z Acceleration – Standard Deviation | $\sigma\left( a_{z} \right)$ |
| Acceleration Norm – Mean | $\left\langle\sqrt{{a_{x}}^{2}+{a_{y}}^{2}+{a_{z}}^{2}} \right\rangle$ |
| Acceleration Norm – Standard Deviation | $\sigma\left( \sqrt{{a_{x}}^{2}+{a_{y}}^{2}+{a_{z}}^{2}} \right)$ |
| X-Y Ratio – Mean | $\left\langle\frac{a_{x}}{a_{y}} \right\rangle$ |
| X-Z Ratio – Mean | $\left\langle\frac{a_{x}}{a_{z}} \right\rangle$ |
| Y-Z Ratio – Mean | $\left\langle\frac{a_{y}}{a_{z}} \right\rangle$ |
| X-Y Ratio – Standard Deviation | $\sigma\left( \frac{a_{x}}{a_{y}} \right)$ |
| X-Z Ratio – Standard Deviation | $\sigma\left( \frac{a_{x}}{a_{z}} \right)$ |
| Y-Z Ratio – Standard Deviation | $\sigma\left( \frac{a_{y}}{a_{z}} \right)$ |
| X-Y Circular Deviation – Mean | $\left\langle1-\left[ {a_{x}}^{2}+{a_{y}}^{2} \right] \right\rangle$ |
| X-Z Circular Deviation – Mean | $\left\langle1-\left[ {a_{x}}^{2}+{a_{z}}^{2} \right] \right\rangle$ |
| Y-Z Circular Deviation – Mean | $\left\langle1-\left[ {a_{y}}^{2}+{a_{z}}^{2} \right] \right\rangle$ |
| X-Y Circular Deviation – Standard Deviation | $\left\langle1-\left[ {a_{x}}^{2}+{a_{y}}^{2} \right] \right\rangle$ |
| X-Z Circular Deviation – Standard Deviation | $\left\langle1-\left[ {a_{x}}^{2}+{a_{z}}^{2} \right] \right\rangle$ |
| Y-Z Circular Deviation – Standard Deviation | $\left\langle1-\left[ {a_{y}}^{2}+{a_{z}}^{2} \right] \right\rangle$ |
| Posture Angle – Mean | $\left\langle\theta\right\rangle$ |
| Posture Angle – Standard Deviation | $\sigma\left( \theta\right)$ |
| Step Count – Mean | $\left\langle s \right\rangle$ |
